# An elderly man with mild encephalitis/encephalopathy with a reversible splenial lesion (MERS) without neuropsychiatric sequelae

**DOI:** 10.1002/ccr3.7219

**Published:** 2023-05-04

**Authors:** Rintaro Fujii, Ryo Yamamoto, Yoshino Inoue, Shunsuke Fukuyo, Takahiro Yamaguchi, Reiji Yoshimura

**Affiliations:** ^1^ Department of Palliative Care and Hemato‐oncology Wakamatsu Hospital of the University of Occupational and Environmental Health Fukuoka Japan; ^2^ Department of Psychiatry University of Occupational and Environmental Health Fukuoka Japan; ^3^ Department of Neurology Wakamatsu Hospital of the University of Occupational and Environmental Health Fukuoka Japan; ^4^ Department of Rheumatology and Diabetology Wakamatsu Hospital of the University of Occupational and Environmental Health Fukuoka Japan

**Keywords:** case report, MERS, mild encephalitis/encephalopathy with a reversible splenial lesion, RESLES, reversible splenial lesion syndrome

## Abstract

Mild encephalitis/encephalopathy with a reversible splenial lesion (MERS) is less common in the elderly, and most have some sequelae. However, even in the elderly, MERS may have a good prognosis, and a specific treatment is not always required.

## Graphical abstract

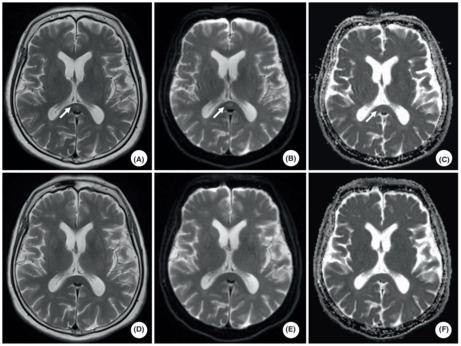

## 1 INTRODUCTION

Reversible lesions can occur in the splenium of the corpus callosum (SCC) due to several factors, including infections, metabolic disorders, and medications.^1^ Garcia‐Monco et al. expressed this as reversible splenial lesion syndrome (RESLES).^2^ In a broad sense, RESLES spectrum includes mild encephalitis/encephalopathy with reversible splenic lesions (MERS). MERS is known as a clinical/radiological syndrome.^3^ Most cases of MERS have been reported in Asia, particularly in Japan.^4^ MERS is characterized by mild impairment of consciousness and neuropsychiatric symptoms, with brain MRI findings and symptoms improving within 1 month of onset. MERS is common in children.^3^ In adults, most cases are reported in young to middle‐aged patients, with few cases occurring in the elderly. SCC lesions in the elderly are usually due to cerebrovascular disease or malignancy. A few minority cases have been reported, but in general, there is little concern about MERS in the elderly.^5^, ^6^, ^7^ Here, we report a case of MERS in an elderly man who improved without neuropsychiatric sequelae.

## 2 CASE PRESENTATION

**FIGURE 1:**
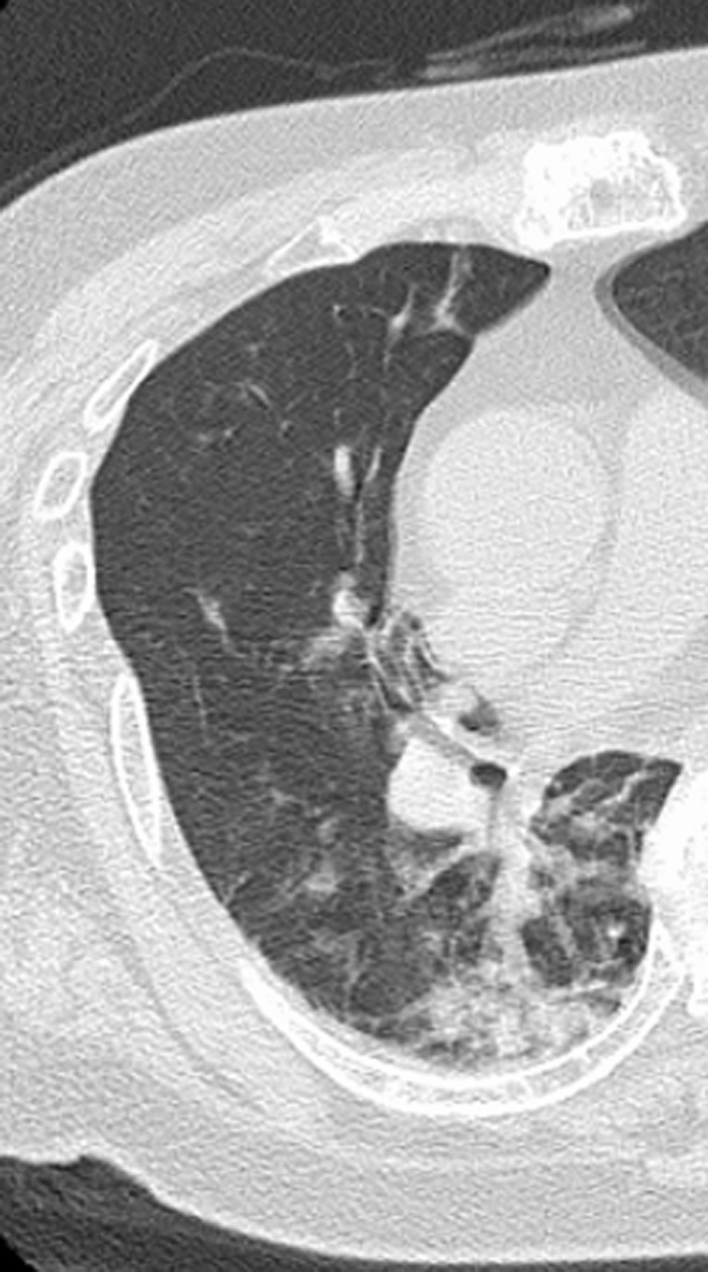
CT of the right lung.

A 74‐year‐old Japanese man was admitted to our hospital's rheumatology department and consulted by the psychiatry department. He lived alone and was independent in his daily life. There was no history or family history of psychiatric disorders. He had no history of thyroid dysfunction or neurological disease but had had untreated bilateral knee pain for 12 years. He was admitted to the rheumatology department for a thorough evaluation of his knee pain. He had tender spots on both knees, and the blood test showed positive biologic markers of rheumatoid arthritis (rheumatoid factor: 136.7 IU/mL, anti‐cyclic citrullinated peptide antibody: 380 U/mL). After these examinations, he was diagnosed with moderate rheumatoid arthritis (Clinical Disease Activity Index: 13). The day after admission, he presented with a fever of 38.5°C. A chest CT showed pneumonia in the right lower lobe (Figure 1). He was diagnosed with pneumonia, and started on treatment with sulbactam/ampicillin at 6.0 g daily. He did not receive drug therapy for rheumatoid arthritis during his admission because the priority was to treat the pneumonia. On the same day, he seemed easily distracted and was not oriented to time and place, and that evening had a confused conversation with a nurse. He was restless, and frequently tried to leave his bed. His restlessness and distraction were observed throughout the day and night. Head CT did not reveal any abnormalities. On the sixth day after admission, he received a psychiatric consultation to determine the cause of his symptoms.

**FIGURE 2:**
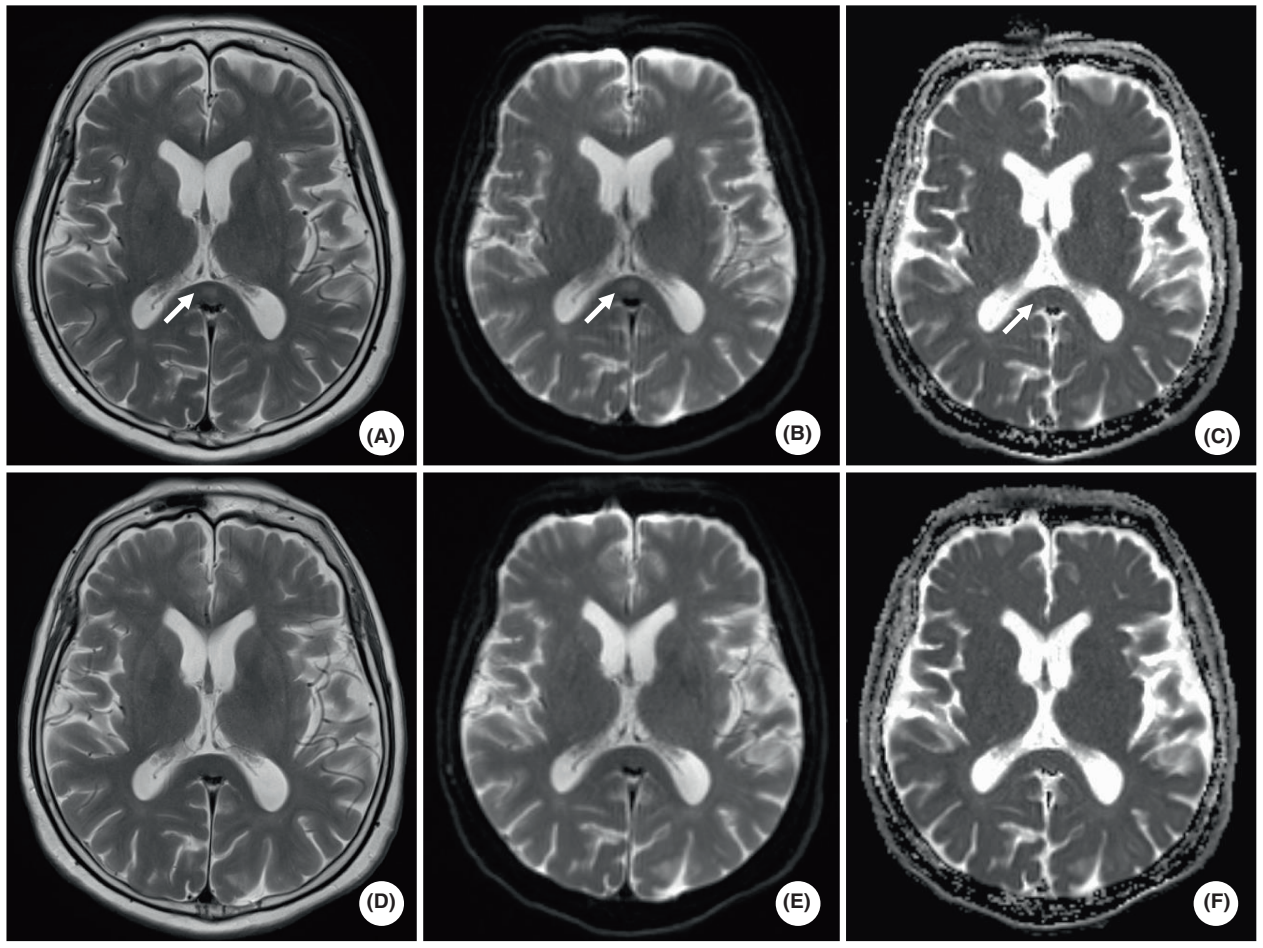
Brain MRI of this case. T2WI (A) and diffusion‐weighted imaging (DWI) (B) reveal hyperintense lesions in the splenium of the corpus callosum. DWI shows low apparent diffusion coefficient values (C). However, the brain MRI 15 days later revealed that the abnormal signal had disappeared in each image (D–F).

He had a mild impairment of consciousness: Glasgow coma scale was 13/15. His body temperature remained approximately 37°C, and his pulse, blood pressure, and respiratory rate were regular. There was no need for oxygen administration. There was memory impairment for events leading up to admission and disorientation to place and time. These signs were accompanied by distractibility and restlessness. His conversations were often derailed, and some delusions (e.g., thinking of being killed in the hospital) were noted. There had been no seizures or loss of consciousness. Nor were there any illusions or hallucinations. He did not use glucocorticoids, benzodiazepines, antiepileptic drugs, or alcohol. Blood tests showed a white blood cell count of 4.9 × 10^9^/L, with a neutrophil fraction of 93.2% and C‐reactive protein of 3.48 mg/L, indicating increased inflammation. No other abnormal findings in electrolytes, renal function, blood glucose level, endocrine hormones, ammonia, or vitamins were identified. Anti‐HIV antibodies were also negative. Electroencephalogram (EEG) recorded 8–10 Hz background EEG activity and no apparent epileptic discharges. On the ninth day after admission, brain MRI showed an elliptical high‐signal lesion in the SCC on diffusion‐weighted imaging and T2‐weighted imaging (T2WI) and a low‐signal lesion in the same area on diffusion restriction on apparent diffusion coefficient mapping (Figure 2A–C). After discussing these findings with the neurologist, it was concluded that the delirium was caused by encephalitis/encephalopathy due to the lesion in the SCC. In addition, the pneumonia in the right lower lobe was considered a significant factor associated with the SCC lesion.

He continued to receive antimicrobial therapy for pneumonia. Because his level of consciousness was only mild impairment, treatment of pneumonia was prioritized, and no glucocorticoids or high‐dose intravenous immunoglobulin (IVIG) were administered. When he was highly agitated, quetiapine 12.5 mg was used. His fever resolved with antimicrobial therapy, and blood tests showed gradual improvement in the inflammation. Chest imaging also showed improvement in the pneumonia. Sulbactam/ampicillin were discontinued on day 15 after admission. During the course of these treatments, his agitation, attention deficit, memory impairment, disorientation, delusion, and impaired consciousness disappeared, and conversation was no longer a problem. Quetiapine was used only on day 7 after admission. A repeat brain MRI 15 days after the previous scan showed that the elliptical lesion in the SCC had disappeared (Figure 2D–F). We diagnosed the cause of the delirium as MERS. After evaluation by the rheumatologist, physical therapy for both knees, and improvement in the delirium, he was discharged on day 41. Since then, he has experienced no delirium or other neuropsychiatric symptoms. A signed informed consent form for publication was obtained from the patient before reporting this case.

## 3 DISCUSSION

Here, we describe the case of an elderly man with MERS, with pneumonia as the primary etiology. The mechanisms for the development of such reversible changes in the SCC are still unclear. However, it has been suggested that transient myelin sheath edema, axonal damage, and oxidative stress may be associated with the pathogenesis of MERS.^3^, ^8^ Tada et al. reported the following clinical findings in MERS: (1) mild disturbance of consciousness, (2) encephalitis/encephalopathy that improves without sequelae within 1 month of onset, and (3) reversible lesions in the SCC that improve within 3 days to 8 weeks of onset.^3^ This case was typical of MERS because each of these characteristics was present. MERS is classified into types I and II, based on whether there are lesions in the brain other than in the SCC.^9^ This case was classified as MERS type I due to the solitary lesion in the SCC. There were no findings of other organic neuropsychiatric disorders other than MERS, and no findings of brain atrophy.

Most MERS cases in the elderly have sequelae or do not improve.^5^, ^6^, ^7^ We hypothesize that the background diseases associated with our present case (rheumatoid arthritis, acute pneumonia) were not as severe as those in previously reported MERS cases in the elderly. While several reports of MERS with hypoglycemia as a factor have shown improvement,^10^, ^11^ there are few cases of MERS in elderly patients caused by an infection, which improved without sequelae. In one case, an elderly MERS patient improved without sequelae after a methicillin‐resistant *Staphylococcus aureus* infection.^12^ Follow‐up MRI was not done; however, it remains unclear whether the improvement in symptoms was accompanied by improvement in the lesions in the SCC. Unlike that report, a repeat brain MRI in our present case showed improvement in the SCC within 1 month of onset. Lesions in the SCC are not limited to MERS cases and include cerebrovascular disease and malignancies.^13^ MERS is more common in young people, while cerebrovascular disease and malignancies are generally more common in the elderly. However, unlike cerebrovascular disease or malignancy, MERS may have a better outcome than these diseases in elderly patients, as in this case. It is therefore crucial to consider that even elderly patients can have MERS and that these patients may have a better prognosis.

Apart from infection, MERS in this patient might also have been caused by his rheumatoid arthritis. His rheumatoid arthritis activity had not changed during hospitalization, however, and rheumatoid arthritis was, therefore, not considered a main factor. In this case, we could not identify the bacteria or virus causing his pneumonia. The antimicrobial therapy was effective, however, suggesting that the patient had bacterial pneumonia. Other factors that may contribute to lesions in the SCC include metabolic disorders, alcohol, and drug‐related factors such as antiepileptic drugs, none of which are applied in this case. Thus, we concluded that MERS was primarily caused by pneumonia.

There were no abnormal findings in the EEG in this case. Although EEG abnormalities can be characteristic of MERS, less than half of the previously reported cases had EEG abnormalities.^14^ The possibility of MERS in the absence of EEG abnormalities must, therefore, be kept in mind. In addition, as we did not perform a lumbar puncture, information from spinal fluid was lacking.

Glucocorticoids and IVIG are sometimes used to treat MERS.^14^ However, several cases have improved without these treatments but rather only by treatment of the underlying disease that caused MERS. Accordingly, these treatments may not always be necessary. Our present case also improved without steroids or IVIG, which supports this suggestion. Treatment guidelines for MERS have yet to be instituted. However, the severity of impaired consciousness and lesions elsewhere in the SCC have been associated with MERS outcomes.^15^ The mild impairment of consciousness in our case and the solitary lesion in the SCC may have been behind the favorable outcome.

## 4 CONCLUSIONS

In conclusion, we note that even the elderly can develop MERS and that cases showing improvement without sequelae do occur.

## AUTHOR CONTRIBUTIONS

**Rintaro Fujii:** Writing – original draft; writing – review and editing. **Ryo Yamamoto:** Writing – review and editing. **Yoshino Inoue:** Writing – review and editing. **Shunsuke Fukuyo:** Writing – review and editing. **Takahiro Yamaguchi:** Writing – original draft; writing – review and editing. **Reiji Yoshimura:** Writing – original draft; writing – review and editing.

## FUNDING INFORMATION

This case report did not receive specific grants from funding agencies in the public, commercial, or not‐for‐profit sectors.

## CONFLICT OF INTEREST STATEMENT

The authors declare no conflict of interest.

## ETHICS STATEMENT

This case report does not involve any studies with human subjects or animals, and therefore, it does not need ethical approval. The confidentiality of the patient was protected and no names nor identifiers were mentioned.

## PATIENT CONSENT STATEMENT

Written and verbal informed consent was obtained from the patient to publish this case report and any accompanying images.

## Data Availability

The data that support the findings of this study are available on request from the corresponding author. The data are not publicly available due to privacy or ethical restrictions.
